# Talking with eye injury patients

**Published:** 2015

**Authors:** Helen Roberts, Daksha Patel

**Affiliations:** Coordinator, Kwale District Eye Care, Mombasa, Kenya.; E-learning Director: International Centre for Eye Health, London School of Hygiene and Tropical Medicine, London, UK.

**Figure:**
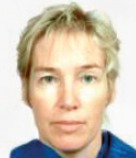
Helen Roberts

**Figure:**
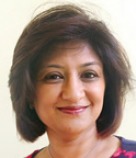
Daksha Patel

Patients with an eye injury are usually in pain and very frightened. They need a gentle, reassuring approach.

Your first task is to assess the general state of your patient. If they are alert and orientated and their general health is good, you can continue to examine them in the eye department. If their immediate general health is at risk, you will have to address this first.

## Immediate communication

It is to be expected that the patient and family members will be anxious. A primary task is to manage this anxiety, which can be achieved by adopting a calm, sympathetic, reassuring and yet authoritative presence. Once the environment is settled, you are then free to enquire gently and clearly what happened and when. Even if emotions are running high, it is imperative that you focus on the assessment and treatment of the injury, particularly if there may be any retained foreign material in the eye.

Where possible, involve the people who have brought the patient to the hospital in the consultation. This is because:

- The patient may take in very little of what is being said and may not be able to explain what happened.
- Caring for the patient is a team effort – and the people accompanying the patient might be needed to instil eyedrops at home, etc.

If you suspect a non-accidental injury you will need to handle this very delicately – focus on the patient and the injury and obtain information without assigning blame or responsibility to anyone. Your hospital should have guidelines for this.

When the immediate pain and injury have been addressed, further communication of a different nature is needed.

## Communication about the prognosis

It is important to be brave and clear but also gentle. Be realistic about the chances of regaining vision or keeping the eye but allow the patient and carers time to accept this. You could say something like the following: ‘The injury is very severe and her/his chances of regaining vision are very poor, but we will do everything we can.’

**Figure:**
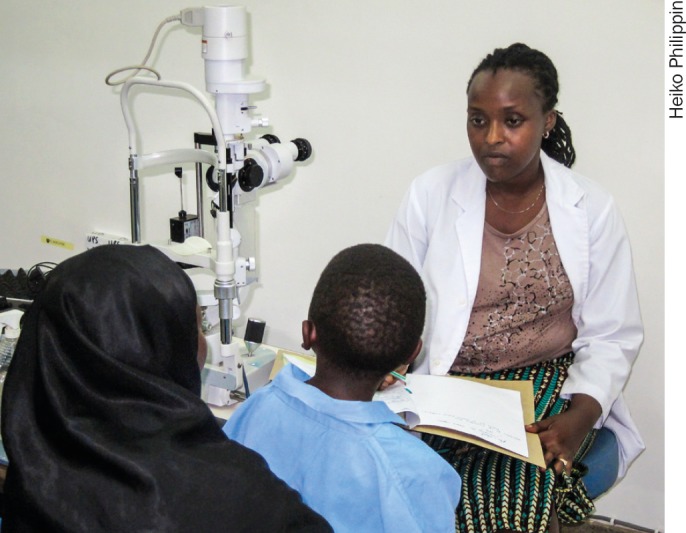
Listening to trauma patients and the people who brought them is an important part of good communication; it will help to reassure patients that they are in safe hands. Children should be kept together with their parents or carers as much as possible.

Even when using the Ocular Trauma Score (see page 44), it can be difficult to predict the prognosis with accuracy in the early stages of management. We advise a cautious approach that avoids any unrealistic optimism. Address what you have worked out is the patient's worst-case scenario.

If you are not sure of the prognosis, we suggest using phrases like the following:

- ‘We don't know yet.’
- ‘Let's see how we get on.’

Blame nothing but the injury. Do not personalise anything or blame any part of previous care, or a delay in referral. It is what it is and the injury is the cause. Bear in mind that everyone in your consultation room might still be in shock following a traumatic incident. Where relevant, offer leaflets and contact details regarding trauma counselling services, support groups, etc.

It is important to give patients and carers time to come to terms with what you have told them about their prognosis. Write down important information and encourage them to ask questions. Verbally repeat the information at least once more before they leave the clinic. Check they understand by asking them to repeat the information back to you and ensure, where appropriate, that they have follow-up appointments and the contact details of who to talk to for further information regarding their prognosis.

## Management and follow-up

Patients may need admission even if they are not having surgery.

If you are happy to send them home, ensure that patients understand how to look after their injured eye.

Make it easy for them to return earlier than asked if something is wrong, orto make contact if they have any questions.

Make sure that you have given the patient and/or the person accompanying them adequate opportunity to ask questions and that you have answered them as best you can.

Explain that long-term follow-up is essential as conditions related to the injury (such as raised IOP or cataract) may develop.

## Prevention of further injury

Evidence suggests that someone who has sustained an eye injury is more at risk of a second eye injury, so prevention should be addressed (see page 51). It may be helpful to discuss this with the patient and carers at a later consultation. Do not hesitate to repeat the information orto contact relevant organisations that can support prevention activities. Keeping a good record of the types of injuries seen in your setting will allow you to develop an appropriate approach for communication about prevention at a local level.

## Legal aspects of injury

Injuries may result in legal action, during which your notes may be required as evidence. As always, write comprehensive notes clearly and avoid any bias or blame. Information that you cannot be sure is fact should be written as: ‘The patient reports that he was injured by a colleague,’ rather than ‘The patient was injured by a colleague.’

In conclusion, patients with injuries do need additional consideration in the way in which you deal with them. The psychological impact of injuries, especially those inflicted deliberately, may be lessened by an empathetic and caring approach from you.

